# The predictability of dynamic preload indices depends on the volume of fluid challenge: A prospective observational study in the operating theater

**DOI:** 10.1097/MD.0000000000012848

**Published:** 2018-10-19

**Authors:** Pierre-Grégoire Guinot, Julien Marc, Bruno de Broca, Thomas Archange, Stéphane Bar, Osama Abou-Arab, Hervé Dupont, Marc-Olivier Fischer, Emmanuel Lorne

**Affiliations:** a Anaesthesiology and Critical Care Department, Dijon University Hospital, Dijon; b INSERM ERI12, Jules Verne University of Picardy; c Anaesthesiology and Critical Care Department, Amiens University Hospital, Amiens; d Anaesthesiology and Critical Care Department, university hospital of Caen, Caen, France.

**Keywords:** fluid challenge, monitoring, esophageal Doppler, stroke volume

## Abstract

This study was designed to assess the association between volume of fluid challenge (FC) and predictability of respiratory variation of stroke volume (ΔrespSV) in the operating theater.

Patients undergoing intermediate/high-risk surgery and monitored by esophageal Doppler monitoring (EDM) were prospectively included. All patients were under general anesthesia and mechanically ventilated. Exclusion criteria were frequent ectopic beats or preoperative arrhythmia, right ventricular failure, and spontaneous breathing. Hemodynamic parameters and esophageal Doppler indices (SV, cardiac output, ΔrespSV) were collected before, after infusion of 250 mL, and after infusion of 500 mL of crystalloid solution. Responders were defined by a >15% increase of stroke volume after FC at each step.

After infusion of a 250 mL FC, 41 patients (32%) were classified as fluid responders (R250). After infusion of a 500 mL FC, 80 patients (63%) were classified as fluid responders (R500). The predictability of ΔrespSV was fair with an area under the curve (AUC) of 0.79 (95% CI 0.71–0.86, *P* < .001) to predict fluid responsiveness with a 250 mL FC. With an AUC of 0.94 (95% CI 0.88–0.97, *P* < .0001), ΔrespSV presented an excellent ability to predict fluid responsiveness with a 500-mL FC.

Predictability of ΔrespSV changed with the volume of fluid infused to assess fluid responsiveness. The accuracy of ΔrespSV was higher with 500 mL than with 250 mL. Bedside studies evaluating the predictability of dynamic preload indices should define fluid responsiveness as a >15% increase of SV in response to a 500-mL FC.

## 1 Introduction

Volume expansion remains a major everyday challenge in the operating theater. While many studies support fluid optimization, other studies have reported deleterious aspects of excessive fluid loading. Over recent years, many published studies have described and promoted dynamic preload indices in the operating theater.^[1–7]^ Dynamic preload indices, such as the respiratory variation of stroke volume (ΔrespSV), are able to predict an increase of cardiac output (CO) following volume expansion (VE). However, some of these studies have reported conflicting results^[2,4–6]^ concerning the predictability of dynamic indices in the operating theater. One reason for these conflicting results could be the lack of standardization of the conditions of fluid challenge (FC), as the majority of studies involved administration of a volume of 500 mL of solution, while other studies administered only 250 mL.^[4–6]^ Taking into account the pharmacodynamics of the solution used for FC, a volume of 250 mL may be insufficient to increase SV over the cut-off used to define fluid responsiveness.^[8–10]^ A number of patients may therefore be classified as nonresponders because of the low SV changes. This lack of standardization prevents reliable comparison of the results of different studies.^[11]^ To date, no study has formally demonstrated that the volume of FC affects the predictability of dynamic preload indices in the same patient. The present study follows on from previous work demonstrating that the definition of fluid responsiveness must be standardized to facilitate harmonization of studies evaluating fluid responsiveness.^[11,12]^

The primary objective of this study was to evaluate the predictability of ΔrespSV according to the volume of fluid infused (250 vs 500 mL). Infusion of 250 mL of fluid may decrease the rate of fluid responders and consequently the predictability of ΔrespSV. The secondary objective was to demonstrate that these results are independent of the type of fluid used (crystalloid /colloid).

## 2 Methods

### 2.1 Ethics

The study objectives and procedures were approved by the local independent ethics committee (Ethics Committee no: RNI2015-33, Comité de Protection des Personnes Nord-Ouest II CHU - Place V. Pauchet, 80054 AMIENS Cedex 1, Chairperson: Thierry Bourgueil) on June 26, 2014. All patients received written information about the study and gave their verbal consent to participate prior to surgery. The present manuscript was drafted in compliance with the STROBE checklist for cohort studies.^[13]^

### 2.2 Patients

A prospective, observational study was conducted in Amiens University Hospital. Patients over the age of 18 years monitored by esophageal Doppler monitoring (EDM), in whom the anesthetist decided to perform VE were included. Indications for VE were: optimization of CO, arterial hypotension (systolic arterial pressure [SAP] below 100 mm Hg and/or mean arterial pressure [MAP] below 70 mm Hg), or compensation of blood loss. Patients with frequent ectopic beats or preoperative arrhythmia, known right ventricular dysfunction and/or pulmonary arterial hypertension, spontaneous ventilation and contraindications to EDM probe insertion were excluded.

Each patient was monitored by pulse oximetry, noninvasive blood pressure monitoring and 5-lead electrocardiogram and underwent balanced general anesthesia. All patients were intubated and ventilated in volume-controlled mode. Tidal volume was adjusted to ideal body weight to obtain 7 to 9 mL/kg (ideal body weight) and ventilatory rate was adapted to maintain end-tidal CO_2_ at 35 to 37 cmH_2_O; positive end-expiratory pressure of 4 to 8 cmH_2_O was applied. The choice of drugs was left to the anesthetist's discretion and comprised either propofol or etomidate for hypnotics and remifentanil or sufentanil for opioids. Anesthesia was maintained with either an inhaled hypnotic (desflurane or sevoflurane) or propofol and the same opioid was used for induction. Depth of anesthesia was monitored by bispectral index of the EEG (BIS, A-2000 monitor, averaging time = 30 seconds; Aspect Medical Systems, Newton, MA). A BIS value between 40 and 60 was considered appropriate. Neuromuscular blockade was systematically induced by rocuronium (0.6 mg/kg) or cisatracurium (0.15 mg/kg). All patients were in the supine position during the study period.

### 2.3 Esophageal Doppler monitoring

The esophageal Doppler probe (CardioQ; Deltex Medical, Gamida, France) was positioned to obtain the optimum signal for descending aortic blood flow velocity. SV and CO were recorded continuously by EDM software (beat by beat) from aortic blood flow velocity. Respiratory variations (Δresp) of EDM values were obtained as previously described, regardless of the respiratory cycle.^[4]^ The respiratory variation of SV (ΔrespSV) was calculated as ΔrespSV = ((SV_max_ − SV_min_)/((SV_max_ + SV_min_)/2) × 100, where SV_min_ and SV_max_ are the minimum and maximum SV values over one respiratory cycle, respectively. All values represented the mean of 5 measurements and were recorded by a physician not involved in the patient's treatment.

The coefficient of variation (CV), precision and least significant change (LSC) for SV were calculated_._ LSC is the smallest SV change that can be considered to be statistically significant, that is, the minimum percentage change between successive measurements that can be considered not due to random error and that therefore represents a real change in SV. The SV’ CV and LSC were determined in all patients at baseline under stable respiratory and hemodynamic conditions as follows: CV = Standard deviation /mean, LSC = CV × √2. Mean CV was 9.5% (1.46), and mean LSC was 13.4% (2.1).

### 2.4 Study protocol

The following clinical parameters were recorded: age, gender, weight, and main diagnosis. In each patient, the choice of fluid bolus was left to the anesthetist's discretion, but crystalloid and colloid boluses were not mixed in individual patients. First, after a 5-minute equilibration period, baseline measurements of HR, SAP, MAP, diastolic arterial pressure (DAP), SV, and CO were obtained. An FC with 250 mL of Ringer lactate over 5 minutes was performed. A second set of measurements (SAP, MAP, DAP, HR, SV, CO) was recorded immediately after the first FC. A second FC with 250 mL of fluid over 5 minutes was then performed. A third set of measurements (SAP, MAP, DAP, HR, SV, CO) was recorded immediately after the second FC. The total infusion time was 10 minutes. Each patient received the same fluid (crystalloid or colloid) at each of the 3 steps. Each patient was included after stabilization of hemodynamic parameters in the absence of any drug injection (sedation, analgesic, vasoactive agents) or changes in ventilatory parameters.

### 2.5 Statistics

A pilot study conducted on 20 patients showed that the area under the curve (AUC) of ΔrespSV was 0.76 for 250 mL, and 0.85 for 500 mL with responder/nonresponder ratios of 34/66% and 70/30%, respectively. We calculated that a sample of 123 patients would be sufficient to demonstrate that the AUC of ΔrespSV is greater after infusion of 500 mL than after infusion of 250 mL (0.85 vs 0.76) for a one-sided alpha risk of 0.05, and 80% power. This calculation was performed according to the methodology proposed by Hanley and McNeil.^[14]^ To take into account the risk of missing data, we planned to include a total of 130 patients.

Nonresponders and responders were defined in terms of the change in SV (expressed as a percentage) after FC. A positive response (fluid responder) was defined as at least 15% increase in SV in response to the FC. This cut-off was chosen in accordance with data from the literature on fluid expansion, and the LSC of SV measurements with EDM.^[11,15,16]^

Pooled data as well as the effects of colloid (colloid group) and crystalloid (crystalloid group) considered separately are reported. The distribution of variables was assessed using D’Agostino-Pearson test. Data are expressed as proportion (percentage), median (25–75th percentiles), or mean (standard deviation), as appropriate. Three groups of patients were defined: nonresponders after infusion of 500 mL (NR), responders after infusion of 250 mL (R250), and responders after infusion of 500 mL (R500). The nonparametric Wilcoxon rank sum test, paired Student *t* test, Mann–Whitney test, Kruskal–Wallis test and analysis of variance (ANOVA) with Bonferroni *post hoc* correction were used to assess statistical significance, as appropriate. A receiver-operating characteristic curve (ROC) was established for ΔrespSV to predict fluid responsiveness after FCs of 250 and 500 mL. The test previously described by DeLong et al was used to compare AUC.

The associations between the volume of fluid infused (ml/kg), the type of fluid infused (colloid/crystalloid), cardiovascular variables (heart rate/respiratory rate, heart rate, SAP, MAP, DAP, SV, CO, ΔrespSV) and fluid responsiveness were assessed using a univariate logistic regression model. Variables with a *P*-value <.10 in the univariate model were included in a multivariate logistic regression model with backward selection. Differences with a *P*-value <.05 were considered statistically significant. IBM SPSS Statistics 22 (IBM) was used to perform statistical analysis.

## 3 Results

About 130 patients were included. Two patients were excluded due to EDM failure. During the study period, no patients were treated by vasopressor, and no patients presented arrhythmia. About 128 patients were analyzed. Indications for surgery were gynecologic cancer surgery (n = 10), visceral surgery (n = 45), urological surgery (n = 45), orthopedic surgery (n = 20), and vascular surgery (n = 8). Patient characteristics are described in Table 1. Mean FC expressed as mL per kg was not significantly different between responders and nonresponders in the overall cohort (Table 1).

**Table 1:**
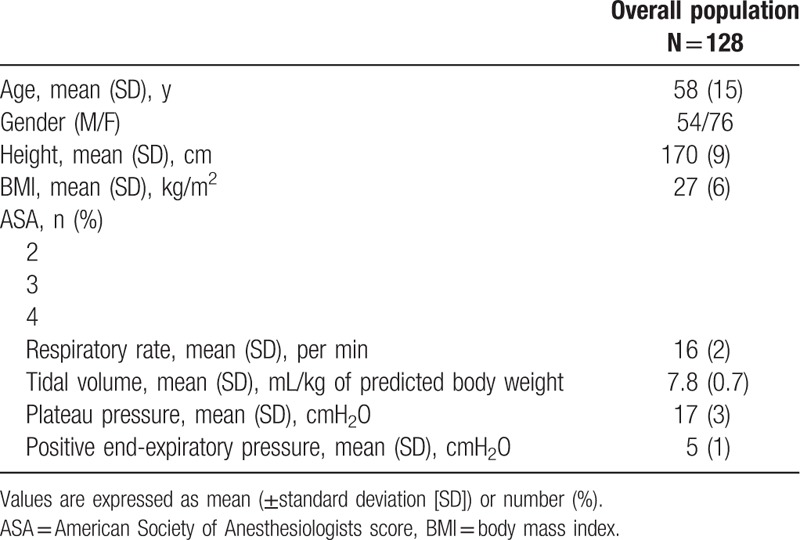
Demographic characteristics of the study population.

### 3.1 Fluid responsiveness

**Figure 1:**
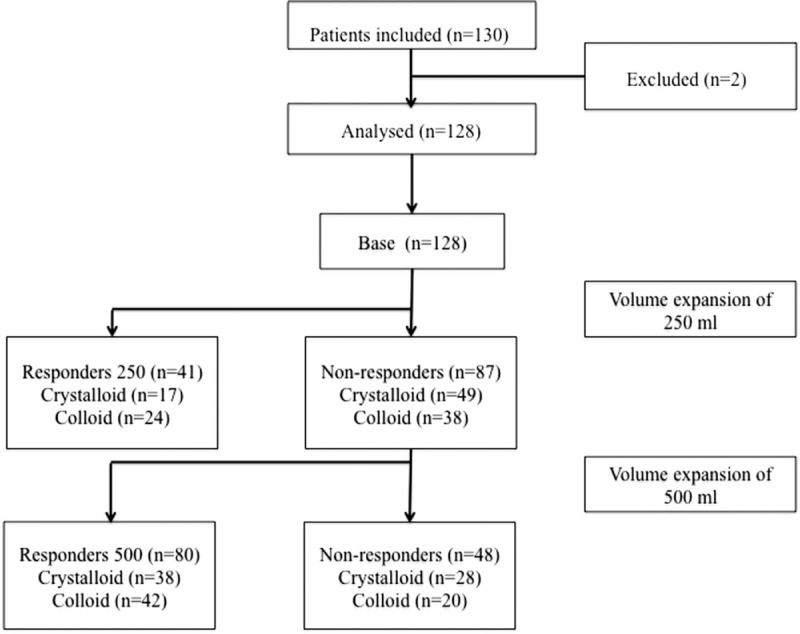
Flow chart.

After the first FC of 250 mL, 41 patients (32%) were classified as fluid responders (R250). After the second FC, 80 patients (63%) were classified as fluid responders (R500). No significant difference was demonstrated between the colloid and crystalloid groups in terms of the prevalence of responders/nonresponders after 250 and 500 mL of FC (*P* > .05) (Fig. 1).

**Figure 2:**
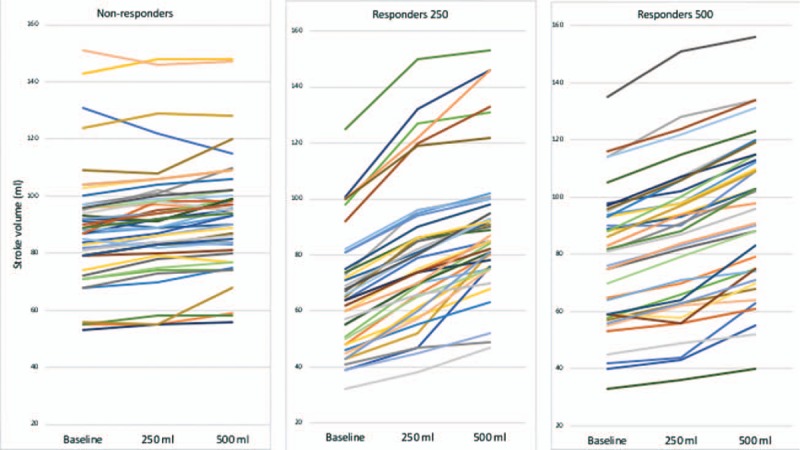
Individual stroke volume values in nonresponders, 250 mL responders, and 500 mL responders at the three steps (baseline, 250 mL, 500 mL).

Baseline CO and SV were lower and ΔrespSV was higher in R250 and R500 than in nonresponders (Table 2). Baseline ΔrespSV and SV was higher in R250 patients than in R500 patients and SV was lower in R250 patients than in R500 patients. FC increased MAP, SV, and CO and decreased ΔrespSV, only in the responder group (R250 and R500) (Tables 2 and 3). The mean increase of SV in response to 250 and 500 mL was higher in R250 patients than in R500 patients (19 ± 5% vs 8 ± 3%, and 40 ± 19% vs 22 ± 8%, *P* < .05). In the crystalloid group, the mean increase of SV in response to 250 and 500 mL was 26 ± 8% and 39 ± 14%, respectively. In the colloid group, the mean increase of SV in response to 250 and 500 mL was 21 ± 8% and 43 ± 22%, respectively (Fig. 2). No significant difference was demonstrated between the crystalloid and colloid groups (*P* > .05).

**Table 2:**
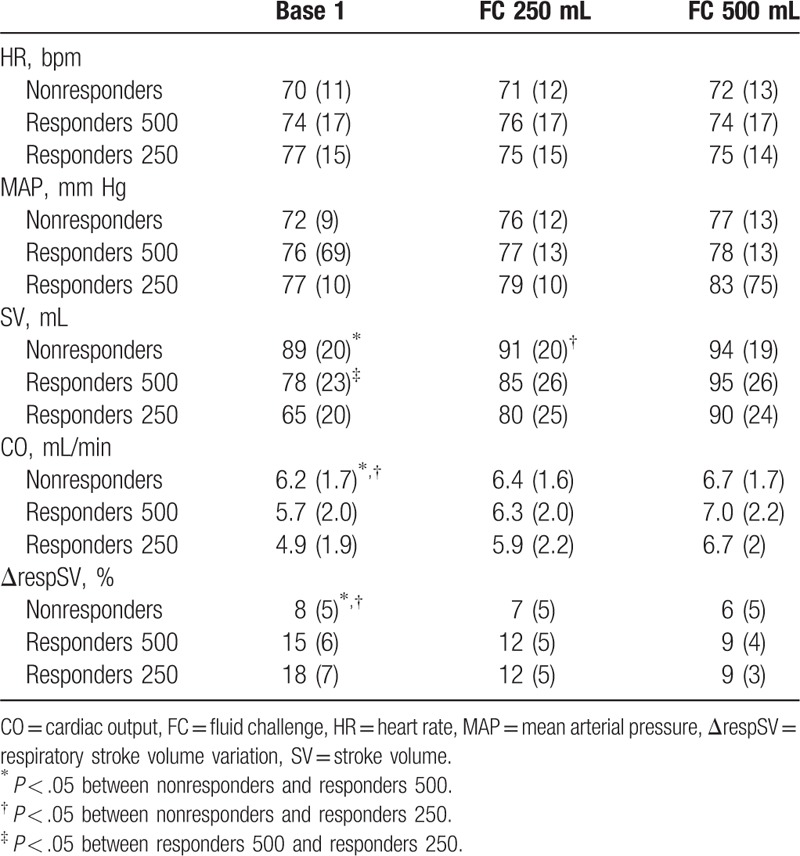
Cardiovascular variables in responders and nonresponders expressed as mean (SD) or median (25–75th percentiles).

**Table 3:**
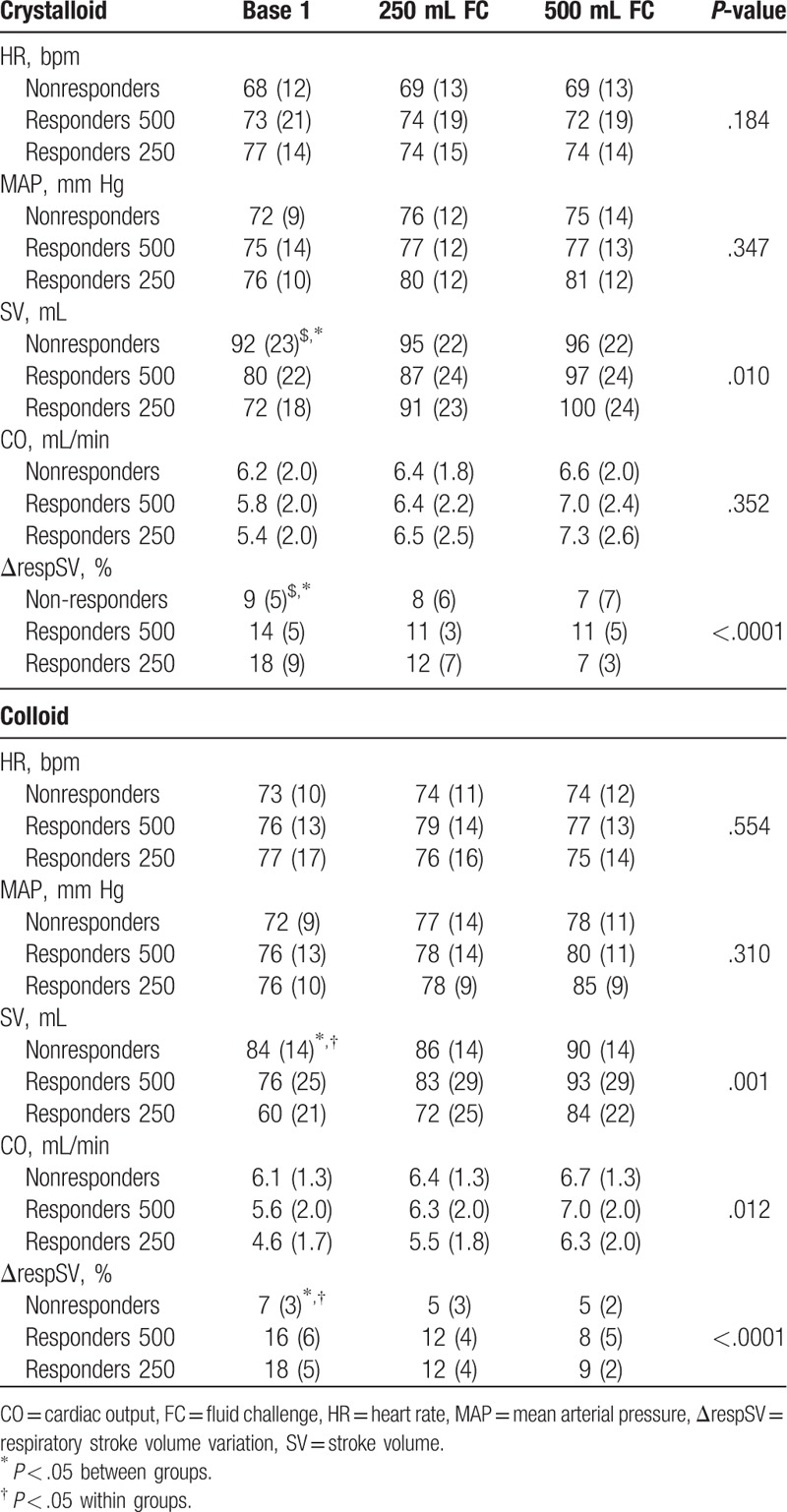
Cardiovascular variables in responders and nonresponders according to type of fluid challenge (crystalloid and colloid groups), expressed as mean (SD) or median (25–75th percentiles).

### 3.2 Predictability according to the volume of FC

The ability of ΔrespSV to predict fluid responsiveness after the first FC of 250 mL was considered to be fair with an AUC of 0.79 (95% CI 0.71–0.86, *P* < .001). The cut-off was 12% with a sensitivity of 78% (62–89%), a specificity of 71% (60–80%), a positive likelihood ratio of 2.62 (1.8–3.8), a negative likelihood ratio of 0.32 (0.2–0.6), a positive predictive value of 54% (41–68%), and a negative predictive value of 87% (78–94%). The gray zone ranged between 10% and 19%.

With a 500-ml FC, ΔrespSV presented an excellent ability to predict fluid responsiveness with an AUC of 0.94 (95% CI 0.88–0.97, *P* < .0001). The AUC of ΔrespSV with a 500-ml FC was greater than the AUC of ΔrespSV to predict fluid responsiveness with a 250-ml FC (*P* < .05). The cut-off was 9% with a sensitivity of 91% (83–96%), a specificity of 79% (65–90%), a positive likelihood ratio of 4.38 (2.5–7.6), a negative likelihood ratio of 0.11 (0.05-0.2), a positive predictive value of 88% (79–94), and a negative predictive value of 85% (71–94). The gray zone ranged between 10% and 12%. No significant difference was demonstrated between the colloid and crystalloid groups (*P* > .05).

On multivariate logistic regression analysis, ΔrespSV was the only factor significantly associated with fluid responsiveness (OR = 1.53, 95% CI: 1.31–1.78; *P* < .0001).

## 4 Discussion

This study shows that the predictability of ΔrespSV changed with the volume of fluid administered to assess fluid responsiveness in the operating theater. ΔrespSV was more accurate with an FC of 500 mL than 250 mL due to the higher rate of fluid responders. This effect appears to be independent of the type of fluid used (colloid vs crystalloid). Fluid responsiveness would be more accurately defined by the increase of SV after a 500-mL FC.

This study demonstrates that up to 50% of fluid-responsive patients could be false-negative with an FC of 250 mL. These results can be explained by several physiologic mechanisms. The first mechanism is related to the patient's venous capacitance. According to Guyton's model, CO is the result of an interaction between cardiac function and venous return. Venous return depends on mean systemic filling pressure (MSFP), venous capacitance, right atrial pressure, and resistance to venous return.^[17]^ In the operating theater, anesthetic agents may increase venous capacitance due to their vasodilator effects.^[18]^ In the absence of vasoactive support, a small volume of FC in anesthetized patients may not be sufficient to increase stressed volume, MSFP and consequently venous return and CO. In the present study, no FC was performed with catecholamine support, in contrast with several published studies that included patients with norepinephrine support.^[19,20]^ Similarly, norepinephrine has been demonstrated to mask preload dependency because it decreases venous capacitance and increases stressed volume.^[21]^ In other words, in patients with high venous capacitance, a large volume of fluid must be infused to significantly increase MSFP, venous return, and CO.

According to the Frank–Starling law, a lower preload is associated with a more marked increase in SV.^[22]^ This correlation is supported by baseline ΔrespSV that was higher in R250 patients than in R500 patients. The increase of SV observed with an FC of 250 mL was also greater in R250 patients than in R500 patients. Because R250 patients had a lower preload than R500 patients, the increase of SV in response to the same volume of FC was greater in R250 patients. These results are in accordance with the shape of the Frank–Starling curve, and may reflect the different indications for FC: correction of hypovolemia, SV optimization. In other words, in patients without profound hypovolemia, physicians need to infuse a significant volume of fluid to significantly increase SV.

No significant difference in the prevalence of R500 patients was observed between the colloid and crystalloid groups. Many studies have evaluated the blood volume-expanding effect of crystalloid and colloid solutions and have demonstrated a greater VE effect of colloids compared to crystalloids.^[23–25]^ In 2010, Trof et al performed a randomized study based on a 90-minute (delta) central venous pressure-guided fluid loading protocol in septic and nonseptic ICU patients.^[24]^ They demonstrated a more marked increase of SV with colloid infusion than with crystalloid infusion at 90 minutes. Nevertheless, a greater volume of colloid was infused. Lanher et al demonstrated an equivalent accuracy of a fixed volume of crystalloid or colloid solution to predict fluid responsiveness during a short-term study.^[7]^ The increase of CO in response to FC was not significantly different between the colloid and crystalloid groups. Studies in the ICU comparing colloids and crystalloids have shown that higher volumes of crystalloids (up to 30%) are usually infused compared to colloids, and that the hemodynamic effect of crystalloids may be less sustained over time.^[24,25]^ In light of all of these results, the volume-expanding property of a solution must not be assessed immediately, but over a longer period of time. No significant difference was observed between colloids and crystalloids in our study, as CO was measured immediately after VE.

In the present study, the pharmacodynamics of the solutions used to assess preload responsiveness may not be one of the major factors impacting the increase of SV (i.e., fluid responsiveness). Because fluid responsiveness depends on the volume of FC, the predictability of dynamic preload indices must be analyzed in light of this factor. These effects may partly explain some of the differences observed between the various studies published in the literature. Several studies that used 250 mL for FC demonstrated the limited ability of dynamic preload indices, even with colloid solutions^[2,6,7]^ to predict fluid responsiveness, while studies that used an FC of 500 mL demonstrated good predictability of dynamic preload indices.^[2,4,12]^ A recent qualitative review highlighted the problem of the different methodologies used in the literature to assess fluid responsiveness.^[10,26]^ Future studies should use 500 mL of fluid to assess the predictability of an indicator.

The potential bias related to this observational study must be considered. First, we did not randomize the indications for FC, the volume of fluid infused (250 vs 500 mL), or the solution used for FC. Baseline characteristics of the study population were not significantly different between responders and nonresponders. Changes in CO were evaluated immediately after FC because the total infusion time was limited to 10 minutes, whereas Aya et al highlighted the fact that maximum CO change was observed one minute after FC.^[27]^ This early assessment of CO, prior to the maximum change, may have led to false-negative results. Nevertheless, the fact that each patient acted as his or her own control would have reduced this effect. Toscani et al demonstrated that the time of assessment of CO change, when it was <10 minutes, did not affect the rate of fluid responders.^[26]^ A randomized study comparing 250 vs 500 mL may have produced the same results. Another limitation of this study was the ED device (CardioQ; Deltex Medical) that does not measure instantaneous aortic diameter. This limitation has been discussed previously.^[3]^ We performed this study in the operating theater, which may differ from the ICU setting in patients with acute circulatory failure.^[27]^ Our results therefore only concern the operating theater, the technique used to assess the predictability of dynamic preload indices, and the volume of FC. This study did not demonstrate whether a 250- or a 500-mL FC would be more effective to improve maximization of CO and decrease postoperative complications, as this was not the objective of the study.

In conclusion, we demonstrated that the predictability of ΔrespSV may depend on the volume of FC administered to assess fluid responsiveness. In the operating theater, ΔrespSV was more accurate with 500 than 250 mL of FC because of the more marked changes of SV. When designing a study to assess the predictability of a preload parameter in the operating theater, fluid responsiveness should be tested with a 500 mL FC.

## Author contributions

PGG conceived, designed and coordinated the study, and drafted the manuscript. JM, EB, BdB, SB and OAA participated in coordination of the study. EL, MOF, and HD participated in coordination of the study and helped to draft the manuscript.

**Conceptualization:** Pierre Gregoire Guinot, Bruno De Broca, Thomas Archange.

**Data curation:** Pierre Gregoire Guinot, Julien Marc, Osama Abou-Arab.

**Formal analysis:** Pierre Gregoire Guinot, Julien Marc, Emmanuel Lorne.

**Funding acquisition:** Julien Marc, Stephane Bar.

**Investigation:** Pierre Gregoire Guinot, Bruno De Broca, Julien Marc, Thomas Archange, Emmanuel Lorne.

**Methodology:** Pierre Gregoire Guinot, Stephane Bar, Marc-Olivier Fischer.

**Project administration:** Pierre Gregoire Guinot.

**Software:** Pierre Gregoire Guinot, Marc-Olivier Fischer.

**Supervision:** Herve Dupont, Emmanuel Lorne.

**Validation:** Pierre Gregoire Guinot.

**Writing – original draft:** Pierre Gregoire Guinot.

**Writing – review & editing:** Pierre Gregoire Guinot.

Pierre Gregoire Guinot orcid: 0000-0002-7019-9727.
